# Characterization of a Novel Revolving Radiation Collimator

**DOI:** 10.7759/cureus.2146

**Published:** 2018-02-02

**Authors:** Georg A. Weidlich, M. Bret Schneider, John R. Adler

**Affiliations:** 1 Radiation Oncology, National Medical Physics and Dosimetry Company, Palo Alto, USA; 2 Department of Neurosurgery, Stanford University School of Medicine, California, USA; 3 Department of Radiation Oncology, Stanford University Medical Center, Stanford, CA, USA

**Keywords:** radiosurgery, radiation leakage, patient whole body dose, iec

## Abstract

Introduction

The ZAP-X is a novel self-contained and first-of-its-kind self-shielded therapeutic radiation device dedicated to brain and head and neck radiosurgery. By utilizing a 2.7-MV linear accelerator and incorporating a design in which a beam stop and major mechanical elements serve a radiation shielding function, the Zap-X does not typically require a radiation bunker. The unique collimator design of the Zap-X is especially critical to the performance of the overall system. The collimator consists of a shielded tungsten wheel oriented with its rotational axis perpendicular to the beam’s central axis; the goal of this design is to minimize radiation leakage. Beam selection is accomplished by rotating the wheel within its tungsten-shielded housing. We investigated radiation leakage from the Zap-X collimator to determine its compliance with internationally accepted standards using direct radiation measurements.

Materials and methods

To measure collimator leakage in the plane of the patient, equidistant measurement stations were defined in a plane perpendicular to the central beam axis (cax) 1 m from this axis (1 m from the radiation focal spot). To measure leakage alongside and adjacent to the accelerator, equidistant measurement stations were located 1 m from the cax along a line parallel to the cax in the plane of the collimator wheel and along a line parallel to the cax 90 degrees offset from the first line of stations.

Results

Radiation leakage emanating from the collimating head of the linear accelerator in the patient plane ranged between 4.0 and 10.4 mR. Radiation along the linear accelerator (1000 R delivered in the primary beam) varied between 1.7 and 6.8 mR and constituted between 0.00017% to 0.00068% of the primary beam. The former radiation originated from X-ray target leakage, while the latter is produced directly by the linear accelerator and both contributed to the overall leakage radiation that would reach a patient.

Discussion

Due to the large diameter of the Zap-X tungsten collimator wheel and the massive Zap-X tungsten cylindrical collimator shield, the overall patient leakage is 0.00104% of the primary beam at a 1-m distance from the beam central axis in the patient plane. Leakage radiation in the patient plane is limited by the International Electrotechnical Commission (IEC) to 0.1% of the total primary radiation. Radiation leakage along the linear accelerator and the collimator housing was determined to be 0.00068% of primary radiation intensity. This leakage value is lower than the 0.1% leakage limit stipulated by IEC by more than a factor of 100.

Conclusions

Typically, an MV radiation therapy system minimizes exposure by utilizing a combination of device and structural shielding. However, the Zap-X has been uniquely designed to minimize the need for structural shielding. Our results indicate radiation leakage from the collimator meets internationally accepted standards as defined by the IEC.

## Introduction

The Zap-X system is a dedicated, self-contained, self-shielded radiosurgery system developed and manufactured by ZAP Surgical Systems, Inc. of San Carlos, California. Utilizing an S-band linear accelerator with a 2.7-MV accelerating potential, this device is designed specifically for stereotactic radiosurgical (SRS) ablation of intracranial and head and neck lesions [1].

The different structural elements of the Zap-X are arrayed to provide the shielding effect that typically is established by the walls, ceiling, and floor of a radiotherapy vault [2]. Most components needed to produce the therapeutic beam such as the radiofrequency power source, waveguide system, beam triggering electronics, and a dedicated beam stop are mounted on or integrated into the primary spherical supporting structure. Furthermore, the patient, who is positioned supine, is enclosed by yet additional scatter shielding consisting of a rotatable iron shell and a shielded, pneumatically elevated door at the foot of the treatment table. By being mounted onto a shielded treatment sphere with dual-axes of independent rotation, the treatment beam from the linear accelerator can be isocentrically positioned across a solid angle of just over 2π degrees, as necessitated for cranial SRS.

A unique collimator design is critical to the overall performance of the Zap-X system. The Zap-X collimator consists of a shielded tungsten wheel oriented with its rotational axis perpendicular to the beam’s central axis; the goal of the design is to minimize radiation leakage. Beam selection is accomplished by rotating the wheel within its tungsten-shielded housing. It should be noted that the substantial shielding of the Zap-X comes at a price that is the overall weight of the unit, but still represents a substantial savings over typical MV vaults and mazes.

Treatment planning with the Zap-X system, especially via its unique collimator, might enable improved radiosurgical dosimetry [3]. Despite the shielded design of the revolving collimator, the approach advocated by Adler, et al. required a significant number of monitor units (MU) of radiation [3]. The possible need for a large amount of MU raises concerns that radiation leakage from the collimator may deliver an excessive dose of radiation to the patient outside of the primary target region. Therefore, this study investigated the radiation leakage from the Zap-X collimator to determine if the leaked radiation amounts comply with the International Electrotechnical Commission (IEC) using internationally accepted measurement standards.

## Materials and methods

Overview of the Zap-X system

**Figure 1:**
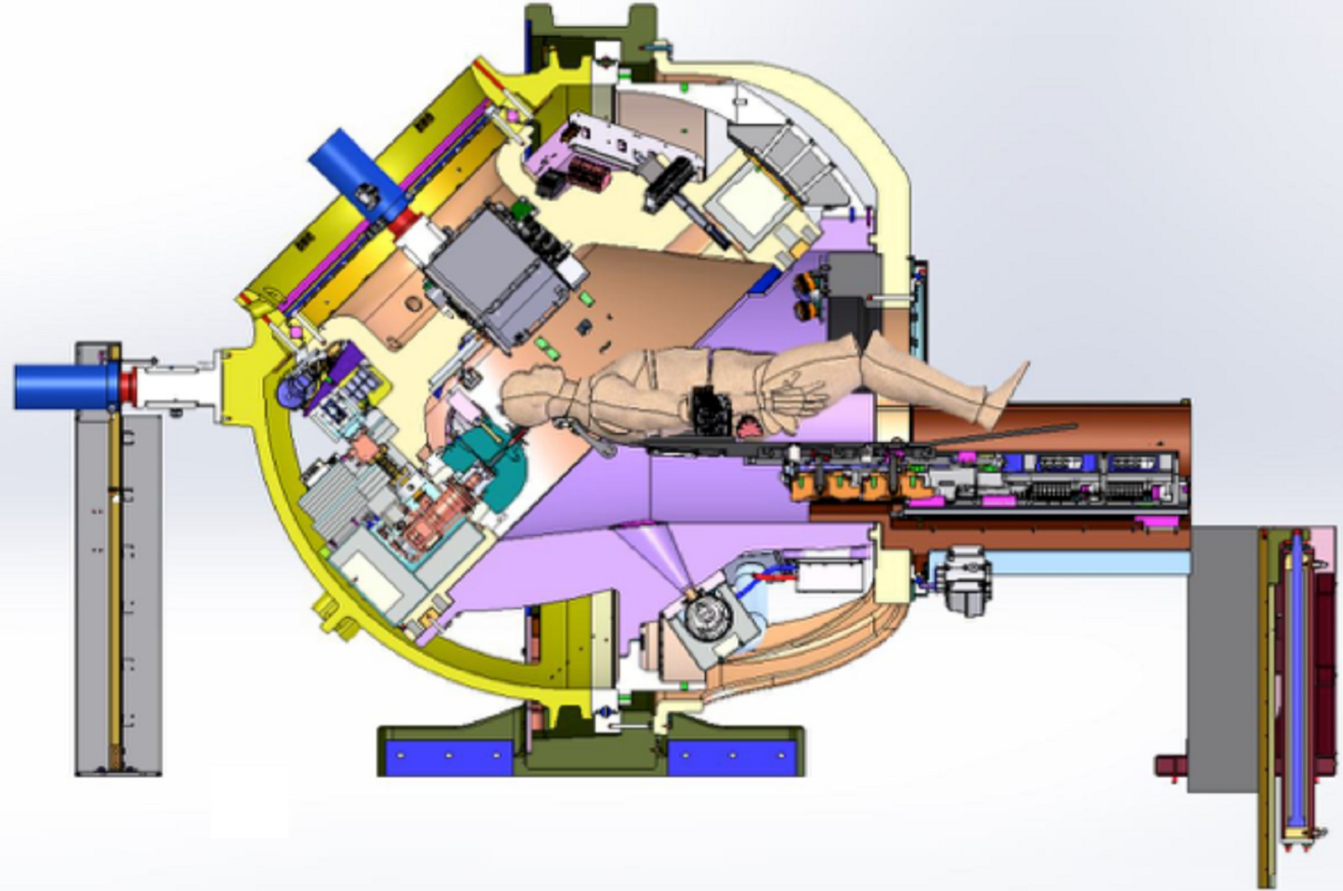
Cross-sectional view of ZAP-X system.

The Zap-X system has a source-to-axis distance (SAD) of 45 cm (Figure 1). Given the short SAD, an isocentric treatment geometry, and the fact that all imaging and shielding components rotate with the beam, the resulting treatment sphere is relatively compact.

Collimator description

**Figure 2:**
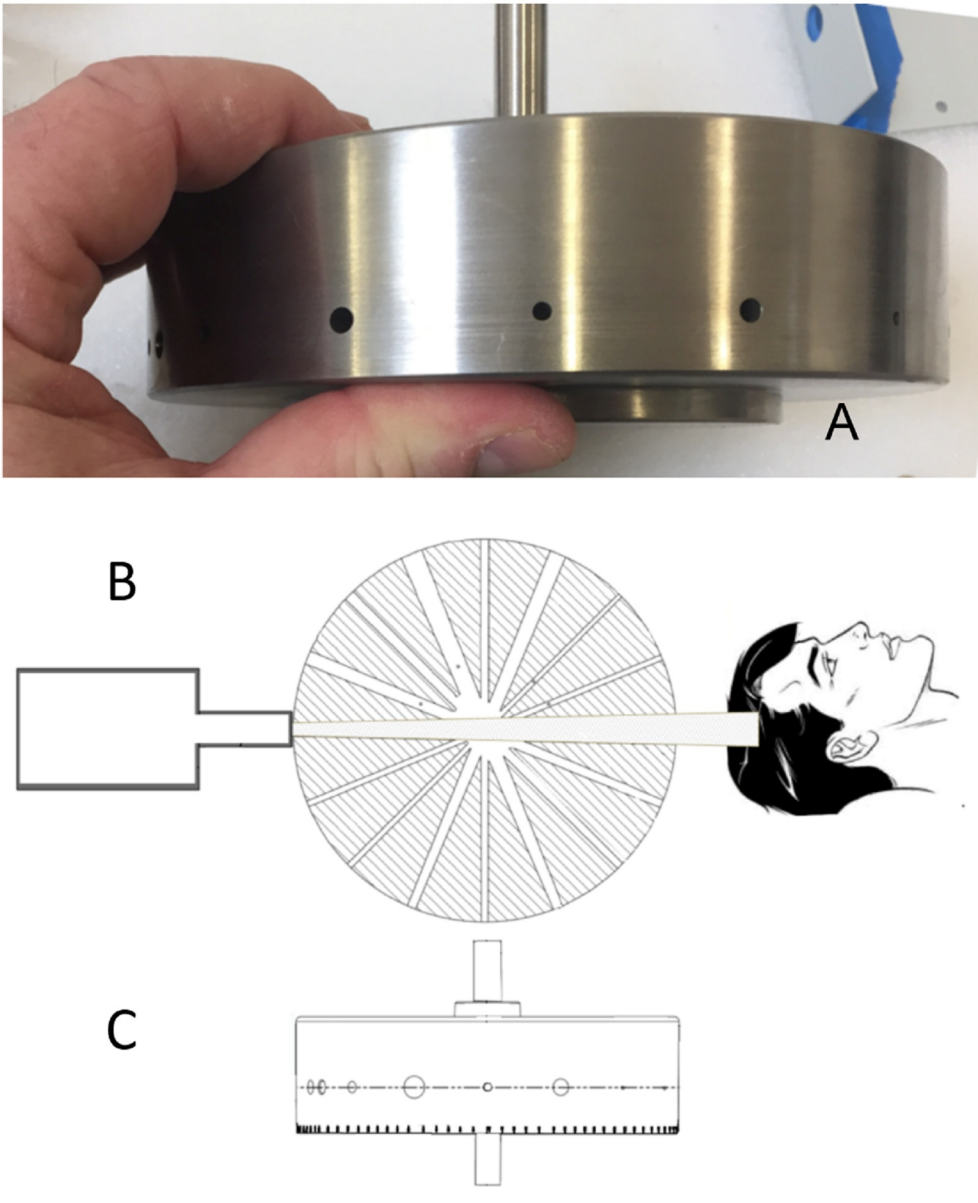
(A) Image of the collimator wheel. (B) Cross-sectional view of the collimator wheel. (C) View along the beam central axis.

**Figure 3:**
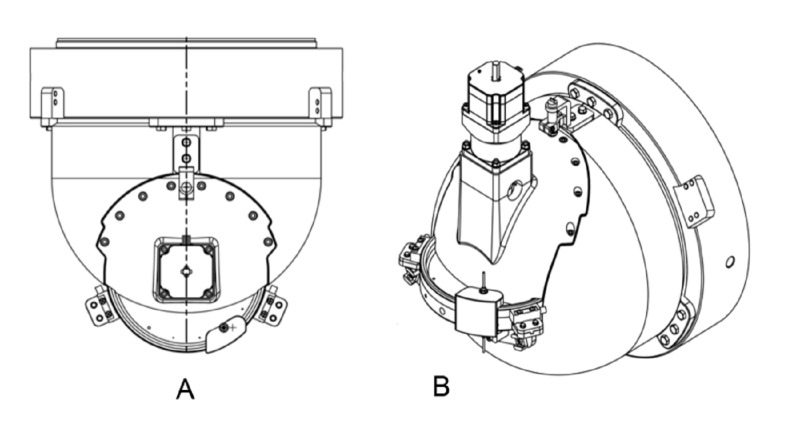
Shielded collimator housing with mounted collimator wheel. (A) Viewed perpendicular to the beam. (B) Viewed oblique to the beam. Collimator wheel is shown directly above the letters A and B in each image.

**Figure 4:**
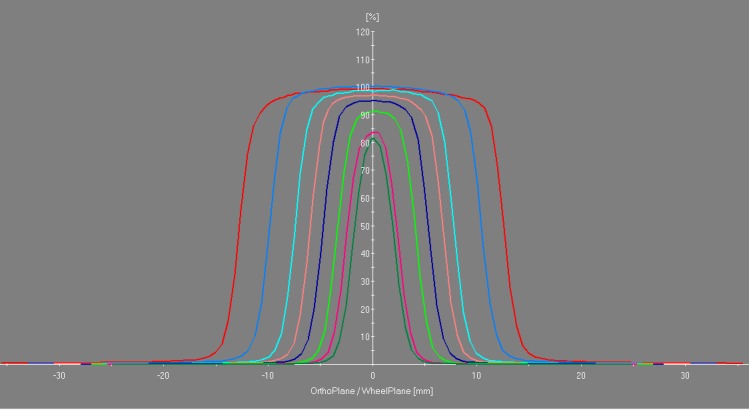
Beam cross profiles at depth of maximum dose for all collimator sizes.

Manufacturers of therapeutic radiation equipment have employed two basic approaches to beam collimation. Most modern radiation oncology machines utilize computer-controlled multi-leaf collimators that enable large, dynamic, custom-shaped beam ports. Other dedicated radiosurgical devices incorporate changeable single-sized circular aperture collimators. The Zap-X system employs a rotating tungsten (W) collimator wheel with divergent small circular paths cut through the wheel. These circular apertures have a range of diameters selected for their practical utility and in accordance with other dedicated cranial radiosurgery system (4.0, 5.0, 7.5, 10.0, 12.5, 15.0, 20.0, and 25.0 mm) as measured at the machine isocenter at maximum build-up depth as the distance between the 50% intensity points with a PTW pinpoint ionization chamber. The distance from the collimator aperture to the isocenter is 28 cm. Previously reported penumbra values for the sequence of Zap-X collimator sizes are displayed in Table 1 [3]. Penumbra is defined at isocenter as the distance between the 80% and 20% intensity points on the beam profile at depth of maximum dose. The wheel is oriented with its rotational axis perpendicular to the beam central axis. Each path of the wheel traverses its diameter at different angular positions to produce the optimal collimator diameter. Figure 2A, 2C shows the collimator wheel image and the beam’s eye view drawing. Figure 2B shows the application of the collimator wheel during the treatment process. The rotating collimator wheel is recessed into a rounded cylindrical-shaped solid tungsten collimator housing, shown in Figure 3. Resulting measured beam profiles are shown in Figure 4.

**Table 1:** Penumbra for various collimator sizes.

Collimator Diameter [mm]	4.0	5.0	7.5	10.0	12.5	15.0	20.0	25.0
Penumbra [mm]	1.8	2.0	2.0	2.2	3.0	3.4	3.4	4.1

All measurement stations were located 1 m from the central beam axis (cax). Measurements in the patient plane were performed at 1 m from the focal spot while measurements along the linac were performed at two groups of stations along a line parallel to the cax in the plane of the collimator wheel and at 90 degrees offset from the first line of stations. Stations are equidistantly spaced at 20-cm intervals. We used a calibrated, large-volume leakage chamber from a Radcal (model #10X6-180, S/N 08-0727, Radcal Corporation, Monrovia, CA) and a survey meter (Fluke Biomedical Model 451 BRYR, S/N 0000003284, Fluke Biomedical, Everett, WA) to measure radiation leakage.

**Figure 5:**
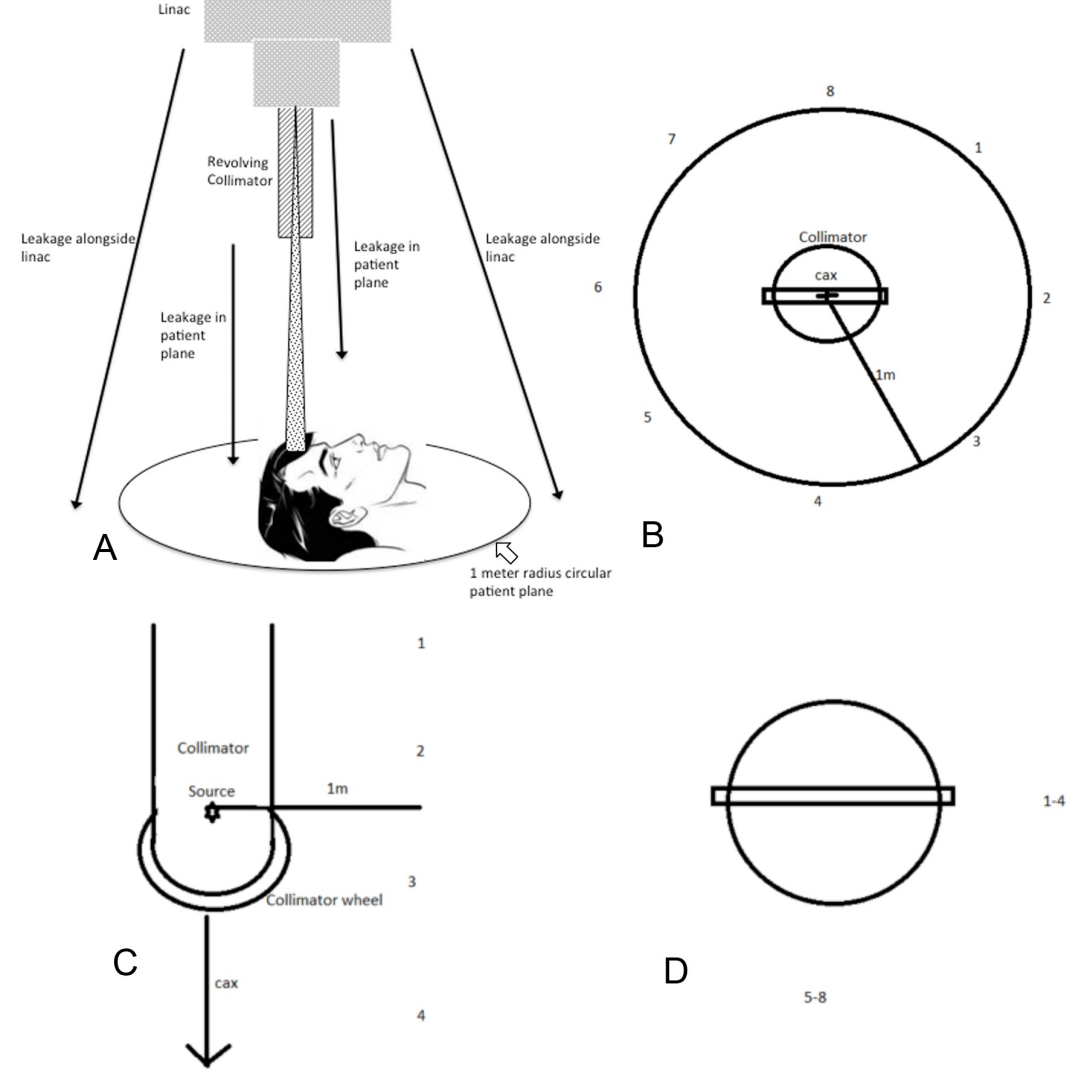
(A) Illustration of patient plane in relation to the patient and linac with collimator. (B) Measurement setup for collimator leakage in the patient plane. The patient plane is defined as a plane perpendicular to the central beam axis (cax) at 1 m distance from the focal spot. In this plane, measurement points are located along a circular line with a radius of 1 m centered on the cax. (C) Measurement setup for leakage along the accelerator in the lateral view. Leakage along the linear accelerator is defined as exposure on a cylindrical surface at a radial distance of 1 m from the cax and centered upon the target. (D) Measurement setup for leakage along the accelerator, beam’s eye view.

Measurement setups for both scenarios are shown in Figure 5A-5D. All measurements were performed with a blank collimator selected, meaning that the collimator wheel was positioned so the beam intercepted the solid tungsten material. The leakage was expressed as a percentage of primary radiation dose measured with a PTW pinpoint ionization chamber.

## Results

It was previously shown that scatter radiation from linear accelerator components reaching the patient is minimal. Measurements of radiation leakage occurring in the patient plane are shown in Table 2 and ranged from 4.0 to 10.4 mR. This radiation inadvertently emanates from the head of the linear accelerator and reaches the patient. Measurements of radiation leakage occurring along the linear accelerator are shown in Table 3. The number of measurements for each station was 5 with a standard deviation of the exposure measurements at 0.45 mR. The percentage of primary radiation was normalized to the largest collimator.

**Table 2:** Radiation leakage measurement results in the patient plane (1000 R delivered in primary beam). Maximum value in bold.

Station #	Reading [mR]	% of Primary
1	5.8	0.00058%
2	5.5	0.00055%
3	5.0	0.00050%
4	4.2	0.00042%
5	4.0	0.00040%
6	4.1	0.00041%
7	7.9	0.00079%
8	10.4	0.00104%

**Table 3:** Leakage measurement results along the linear accelerator (1000 R delivered in primary beam). Maximum value in bold. This radiation originates from the X-ray target and contributes to the overall scatter radiation reaching the patient.

Station #	Reading [mR]	% of Primary
1	2.0	0.00020%
2	4.3	0.00043%
3	6.8	0.00068%
4	4.9	0.00049%
5	1.7	0.00017%
6	6.0	0.00060%
7	6.1	0.00061%
8	3.7	0.00037%

## Discussion

Collimator leakage is important because it represents radiation that may be absorbed by the patient in areas other than the target. Our goal was to determine whether the collimator design of the Zap-X system would meet well-established IEC standards for acceptable levels of radiation leakage.

Leakage radiation in the patient plane is limited by IEC Standard 60601-2-1, 2014 to 0.1% of the primary radiation [4]. Due to the large diameter of the Zap-X tungsten collimator wheel and the massive Zap-X tungsten cylindrical collimator shield, the overall radiation leaked to the patient is 0.00104% of the primary beam at 1 m from the beam central axis in the patient plane. As this leakage radiation is expected to be absorbed by a large portion of the patient’s body distant from the treatment volume, the whole body dose from collimator leakage in patients treated with the Zap-X is expected to be very low.

Radiation leakage alongside the linear accelerator was determined to be 0.00068% of the primary radiation intensity. This leakage value is lower than the 0.1% leakage limit stipulated by IEC by a factor of over 100. While this leakage does not reach the patient directly, it contributes to the overall scatter radiation inside the treatment sphere and is partially absorbed by the patient. Because leakage alongside the linear accelerator and the collimator housing handily exceeds international standards, it is projected that low amounts of radiation scatter will be absorbed by the patient.

The Zap-X collimator was designed to achieve rapid dose fall-off at the beam edge, thereby minimizing penumbra. Beam penumbra is the horizontal distance between the 80% and 20% dose or the intensity values of a beam profile measured at the treatment depth and normalized to its central axis dose value. The Zap-X system has a penumbra of 1.8 mm to 4.1 mm at the depth of maximal dose. This means that the Zap-X system is designed to deliver more of the radiation to the intended target and reduces radiation reaching off-target areas of the brain, thus supporting the anticipated radiation safety associated with treatment. Furthermore, the short SAD reduces beam spread, which also helps reduce off-target radiation.

Low collimator leakage contributes to the improved delivery of complex yet highly conformal radiosurgical treatments to oddly shaped targets, which, by nature, require both many beams and MUs.

This study exclusively addresses collimator radiation leakage while the authors recognize that within-patient internal scatter radiation is the other large contributor to the out-of-field whole body patient dose received during Zap-X radiosurgery delivery. As the collimator leakage was determined to be relatively low compared to IEC standards, this in-patient scatter radiation should be investigated in future studies and is expected, relative to the small direct leakage, to constitute a major component of the whole body dose received by the patient.

## Conclusions

Our findings indicate radiation leakage from the Zap-X collimator meets internationally accepted standards as defined by IEC Standard 60601-2-1, 2014. As the standard is 0.1% and the Zap-X leakage is approximately 0.001%, the standard is exceeded by a factor of 100. The ultimate benefit is that more radiation is delivered to the intended target and less radiation is received by non-target tissues.
